# Derivation and Validation of an Automated Search Strategy to Retrospectively Identify Acute Respiratory Distress Patients Per Berlin Definition

**DOI:** 10.3389/fmed.2021.614380

**Published:** 2021-03-11

**Authors:** Xuan Song, Timothy J. Weister, Yue Dong, Kianoush B. Kashani, Rahul Kashyap

**Affiliations:** ^1^Division of Pulmonary and Critical Care Medicine, Department of Medicine, Mayo Clinic, Rochester, MN, United States; ^2^Intensive Care Unit, Liaocheng Cardiac Hospital Affiliated to Shandong First Medical University, Shandong, China; ^3^Intensive Care Unit, DongE Hospital Affiliated to Shandong First Medical University, Shandong, China; ^4^Anesthesia Clinical Research Unit, Mayo Clinic, Rochester, MN, United States; ^5^Department of Anesthesiology and Perioperative Medicine, Mayo Clinic, Rochester, MN, United States; ^6^Division of Nephrology and Hypertension, Department of Medicine, Mayo Clinic, Rochester, MN, United States

**Keywords:** automation, electronic health records, acute respriatory distress syndrome, adult, ICU

## Abstract

**Purpose:** Acute respiratory distress syndrome (ARDS) is common in critically ill patients and linked with serious consequences. A manual chart review for ARDS diagnosis could be laborious and time-consuming. We developed an automated search strategy to retrospectively identify ARDS patients using the Berlin definition to allow for timely and accurate ARDS detection.

**Methods:** The automated search strategy was created through sequential steps, with keywords applied to an institutional electronic medical records (EMRs) database. We included all adult patients admitted to the intensive care unit (ICU) at the Mayo Clinic (Rochester, MN) from January 1, 2009 to December 31, 2017. We selected 100 patients at random to be divided into two derivation cohorts and identified 50 patients at random for the validation cohort. The sensitivity and specificity of the automated search strategy were compared with a manual medical record review (gold standard) for data extraction of ARDS patients per Berlin definition.

**Results:** On the first derivation cohort, the automated search strategy achieved a sensitivity of 91.3%, specificity of 100%, positive predictive value (PPV) of 100%, and negative predictive value (NPV) of 93.1%. On the second derivation cohort, it reached the sensitivity of 90.9%, specificity of 100%, PPV of 100%, and NPV of 93.3%. The strategy performance in the validation cohort had a sensitivity of 94.4%, specificity of 96.9%, PPV of 94.4%, and NPV of 96.9%.

**Conclusions:** This automated search strategy for ARDS with the Berlin definition is reliable and accurate, and can serve as an efficient alternative to time-consuming manual data review.

## Introduction

Acute respiratory distress syndrome (ARDS) is an acute inflammatory lung injury which occurs in the absence of cardiogenic pulmonary edema and leads to increased pulmonary vascular permeability, increased extravascular lung water, and loss of aerated lung tissue (1). Estimates of the hospital-based incidence of moderate to severe ARDS vary from 1.6 to 7.7% of all intensive care unit (ICU) admissions and 8.0–19.7% of all ventilated patients (2–5). Additionally, the reported population-based incidence of ARDS varies from 10.1 to 86.2 cases per 100,000 person-years (6–9). Overall mortality associated with ARDS is ~40%, according to the most recent observational studies (10, 11). Currently, there is no disease-specific pharmacotherapy to increase survival, and ARDS management remains supportive; therefore, the identification of ARDS with the Berlin definition in the ICU is critical, not only to identify the cases early and start primary and secondary prevention strategies but also to identify ARDS cases for potential clinical prospective studies.

Traditional paper charts have been rapidly replaced by electronic medical records (EMRs). The use of EMRs as a tool to reduce cost and improve safety has been increasing over the years in both clinical practice and health care research (12). For research, EMRs has moved medicine into the era of “big data,” where an unprecedented amount of information can allow for evaluation and identification of risk factors at the population level. ARDS is often not documented in addition to respiratory failure terms. ICD-9 terms are not specific to ARDS and often code to non-specific conditions such as “respiratory distress;” therefore, it is difficult to identify ARDS cases for clinical study. A manual chart review for ARDS diagnosis could be laborious and time-consuming, so the effective and accurate use of EMRs, structured search strategies, and data capturing to identify cases are critical. In retrospective studies related to ARDS, an automated search strategy would be useful to identify cases in a timely fashion with high precision. Other similar search strategies from our team to identify sepsis, post-operative complications, acute kidney injury, and extubation failure have been developed and validated (13–16). These investigators found that by using such electronic search strategies, they were able to achieve high sensitivity and specificity in detecting patients with the syndromes and complications mentioned above.

In this study, our primary aim was to develop and validate a reliable electronic search strategy to identify cases with ARDS with the Berlin definition. Our secondary aim was to compare the sensitivity and specificity of our automated search strategy with a reference standard generated by a comprehensive, manual review of the medical record.

## Materials and Methods

This study was approved by the Mayo Clinic Institutional Review Board (IRB) for the use of existing medical records of patients who gave prior research authorization.

### Study Population

**Figure 1:**
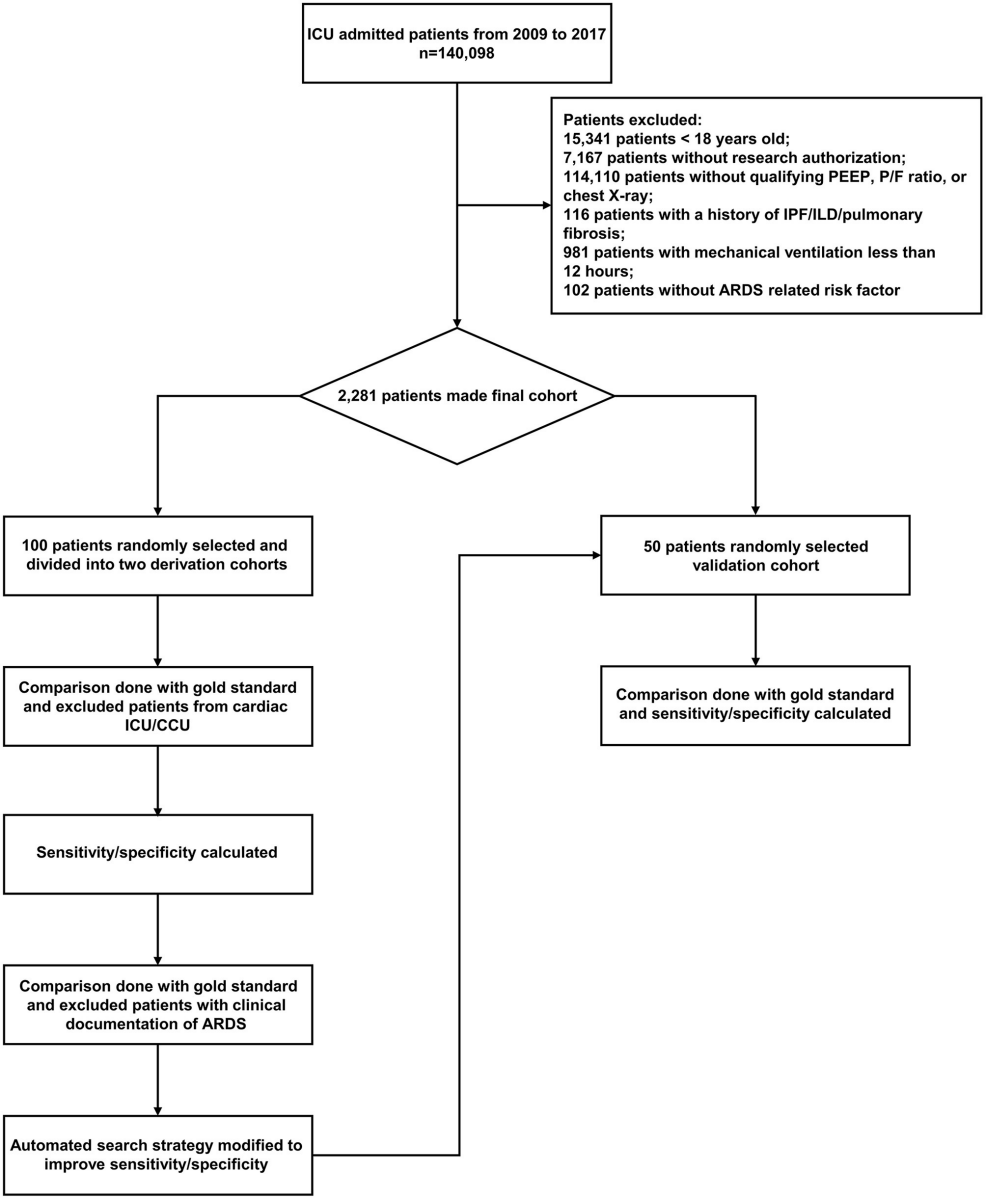
Flow chart of study derivation and validation cohorts. ARDS, acute respiratory distress syndrome; IPF, idiopathic pulmonary fibrosis; ILD, interstitial lung disease; ICU, intensive care unit; CCU, cardiac care unit; PEEP, positive end expiratory pressure; P/F ratio, partial pressure of oxygen to fraction of inspired oxygen ratio.

The study population consisted of all patients admitted to the ICUs at Mayo Clinic in Rochester, MN from January 1, 2009 to December 31, 2017. Among this population, two groups of 50 patients were selected by purposeful sampling for derivation. This random purposeful sampling was done to include a random number of ARDS patients for higher yield. Both the manual reviewer and gold standard were blinded to the results of this sampling.We used separate revision cohorts to be able to test each change in the search strategy in a different group of patients, and, therefore, be able to optimize the search strategy. An additional cohort of 50 random patients was selected for the validation cohort (Figure 1).

We used the Berlin definition of ARDS criteria (1). The Berlin definition partitions patients by PaO_2_/FiO_2_ ratio into mild (PaO_2_/FiO_2_ 200–300), moderate (PaO_2_/FiO_2_ 100–199), and severe ARDS (PaO_2_/FiO_2_ <100) and no longer includes the term “acute lung injury.” This definition also clarifies several areas, including onset, which must be within 1 week of a known clinical insult or new or worsening respiratory symptoms; chest imaging, which must include bilateral opacities that are not fully explained by effusions, lobar collapse, or nodules; and origin of edema, which cannot be fully explained by cardiac failure or fluid overload and must be objectively evaluated (e.g., by echocardiography) if no apparent predisposing factor for ARDS is present. The Berlin definition also sets a minimum positive end-expiratory pressure (PEEP) level of 5 cm H_2_O during PaO_2_/FiO_2_ determination because it has been recognized that changes in PEEP may reclassify patients from the current definition of ALI to ARDS (1).

### Manual Data Extraction Strategies

A manual review of patient EMRs was used for data extraction and ARDS adjudication. The manual reviewer was a practicing clinician, who reviewed the electronic medical charts with all available information. The reviewer assessed all included patients to identify patients who had ARDS per Berlin definition. The reviewer was not involved in the development or utilization of the automated electronic search strategy. Hence, the reviewer was not aware of the results of the automated search strategy.

We used the definition of Berlin ARDS criteria for manual chart review, and defined ARDS based on the presence of both of the following conditions simultaneously: (1) patients with PaO_2_/FiO_2_ ratio <300, PEEP ≥5 cm H_2_O, bilateral infiltrate or edema per chest X-ray, and (2) the presence of at least one risk factor for ARDS (i.e., sepsis/septic shock, pneumonia, pancreatitis, trauma, aspiration, multiple transfusion, drug overdose, and shock). We used the final adjudicated ARDS cases based on this process as the gold standard for the study.

### Automated Electronic Search Strategy

Data were used from Mayo Clinic ICU DataMart and Unified Data Platform, which are extensive data warehouses containing a near real-time normalized replica of Mayo Clinic's EMRs. These databases contain patient information, their laboratory test results, and clinical and pathological information from sources within the institution and have been previously validated (17, 18). Ventilator parameters (such as PEEP) were captured from the ventilators.

**Figure 2:**
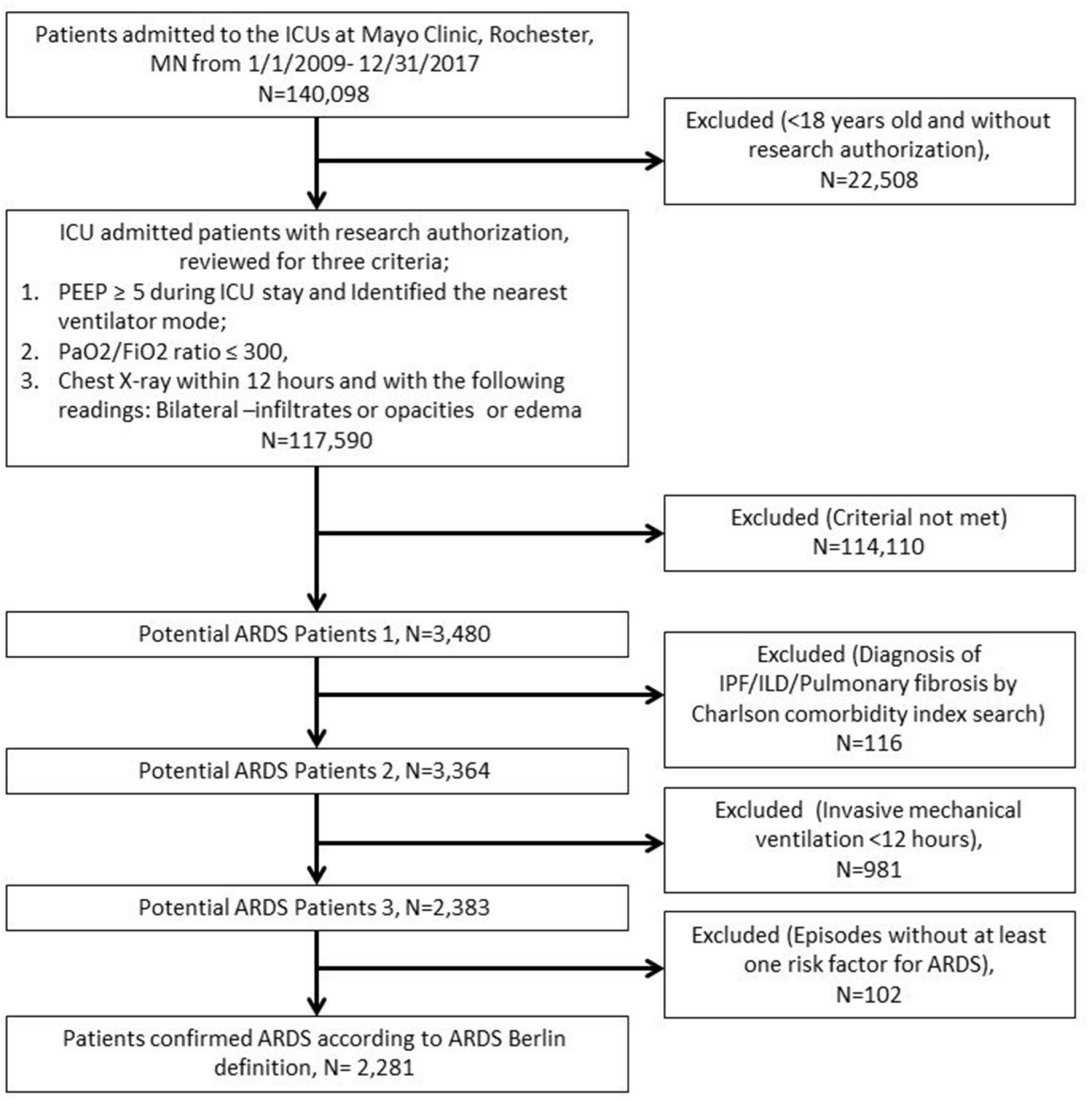
The automated electronic search strategy for identifying ARDS patients per Berlin definition.

The automated electronic search strategy for identifying ARDS patients per Berlin definition was developed in the following sequential steps (Figure 2). First, patients were excluded who did not provide research authorization, along with those <18 years old. Second, ARDS patients were identified according to the following criteria: (1) PEEP ≥5 during the ICU stay (this is “time zero”) and identified the ventilator mode nearest (limited to ICU areas—procedures excluded); (2) Partial pressure of oxygen to fraction of inspired oxygen (PaO_2_/FiO_2_) ratio ≤300, P/F ratios were first established based on matched PaO_2_ and FiO_2_ from labs. If FiO_2_ labs were missing, FiO_2_ from vital signs within ±15 min (nearest) the PaO_2_ value were used; (3) Chest X-ray within 12 h and review radiology report for any of the following combinations: bilateral infiltrates, bilateral opacities, or bilateral edema. If one of them was present, it was considered a positive radiology report. If all 3 criteria in second step were positive, then they were classified as potential ARDS patients. Third, patients with diagnosis of Idiopathic Pulmonary Fibrosis (IPF)/Interstitial Lung Disease (ILD)/pulmonary fibrosis were excluded by Charlson comorbidity index search. Fourth, patients with invasive mechanical ventilation <12 h were excluded, and the duration of mechanical ventilation was searched according to our previously published algorithm (19). Fifth, ARDS risk factors were searched for (i.e., sepsis/septic shock, pneumonia, aspiration, pancreatitis, trauma, drug overdose, shock, and multiple transfusions), and the search strategy for each risk factor was defined. Finally, patients with cardiogenic pulmonary edema, cardiogenic shock, and positive acute decompensated heart failure during ICU admission were excluded, and patients with cardiogenic pulmonary edema risk factors were also excluded by clinical note searches (20), and these risk factors include history of Coronary Heart Disease (CAD), chronic heart failure (CHF), and New ST-changes/Left bundle branch block (Electrocardiography query within ±24 h of 1st PEEP ≥5). The automated search algorithms were validated in comparison with the gold standard obtained by manual review.

### Statistical Analysis

For automation process, the only applicable analysis is sensitivity and specificity for a nominal variable (ARDS, yes/no). The sensitivity and specificity of the search algorithms were calculated by comparing the results to the gold standard obtained by manual review of the charts. We used JMP Pro 14 statistical software (SAS Institute Inc., Cary, NC, USA). *P*-values 0 < 0.05 were considered statistically significant.

## Results

Between January 1, 2009, and December 31, 2017, 140,098 adult patients with research authorization were admitted to the participating ICUs, and 2,281 patients met the ARDS Berlin definition according to the automated search strategy. A total of 100 patients were chosen after purposeful sampling to be included in the two derivation cohorts, and an additional 50 patients were selected for the validation cohort.

The automated search strategy identified ARDS patients with a sensitivity of 91.3%, specificity of 100%, positive predictive value (PPV) of 100%, and negative predictive value (NPV) of 93.1% in the first derivation cohort (Table 1). Disagreements between the automated search strategy and the manual review were observed in 2 patients in this data subset, both false negatives. In one of the cases, ARDS was missed by the digital algorithm as PaO_2_/FiO_2_ ratio, and chest X-ray were not found, while in the other case sepsis developed >72 h after ICU admission. In the second derivation cohort (Table 1), the automated search strategy reached a sensitivity of 90.9%, specificity of 100%, PPV of 100%, and NPV of 93.3%. Disagreements between the automated search strategy and the gold standard occurred in 2 patients, both false negatives. The reasons for these false-negative cases were identical to those in the first derivation cohort. The manual vs. automated cohorts had same baseline characteristics as they were exact same cohorts (data not shown).

**Table 1:** Automated search strategy sensitivity and specificity for ARDS per Berlin definition.

	**ARDS per Berlin definition**			
	**Sensitivity (%)**	**Specificity (%)**	**PPV (%)**	**NPV (%)**
Derivation cohort 1	91.3	100	100	93.1
Derivation cohort 2	90.9	100	100	93.3
Validation cohort	94.4	96.9	94.4	96.9

*ARDS, acute respiratory distress syndrome; PPV: positive predictive value; NPV, negative predictive value*.

In the validation cohort, the automatic search strategy yielded a sensitivity of 94.4%, specificity of 96.9%, PPV of 94.4%, and NPV of 96.9% (Table 1). Disagreements between the automated search strategy and the reference standard occurred in 2 patients, both false negatives. One case was due to missing PaO_2_/FiO_2_ ratio and chest X-ray, and the other case was because the patient used home Bilevel Positive Airways Pressure (BiPAP), and thus PEEP was not electronically recorded.

## Discussion

In this study, we demonstrated an automated search strategy for ARDS that could effectively and accurately identify patients based on the accepted clinical definition (i.e., Berlin definition, among ICU patients). Several previously automated search strategies have been described in the literature (13–15, 21, 22); however, to date, the Berlin definition has not been used as a digital signature of ARDS patients.

As EMR utilization continues on an upward trajectory, the volume of available information to generate and validate digital signatures of different clinical syndromes in ICUs has grown. The accumulation of vast amounts of data provides opportunities to improve the processes of care and treatment. Manual chart review for ARDS diagnosis would likely be laborious and time-consuming; considering the significant shortage of human resources in clinical investigations, there seems to be a vital need to use EMRs for syndrome detection. Traditional ICD code searches for such conditions may not be completely sensitive or specific (18, 23), and changes in coding guidelines make them even less reliable. Thus, the development of automated search strategies can prove useful for clinical and research purposes.

Our study has several strengths. To our knowledge, this is the first study regarding the development and validation of an automated search strategy within EMRs for the identification of ARDS patients per Berlin definition. It is a valuable contribution in that it allows for a quick and reliable way to identify cases of ARDS retrospectively, which will ultimately enable pragmatic research on large cohorts of patients using existing EMRs. Using automated search strategies overcomes the barrier of time-consuming manual review and mitigates human errors that occur during manual data extraction. This electronic signature provides strong support for educational and research activities and demonstrates a simple yet effective method that can be applied to other clinical conditions.

Several limitations of our study should be acknowledged. First, the accuracy of the EMR depends on the precision of written clinical notes. As with manual chart review, we assumed clinical documentation is accurate, while errors in the documentation are possible. In our institution, periodic quality checks on clinical notes are done with frequent audits. Therefore, we believe the impact of documentation errors in this digital signature is minimal. Secondly, this is an automatic search strategy to retrospectively identify ARDS patients per the Berlin definition. It cannot identify these patients in real time, but it lays a foundation for the development of ARDS software to identify ARDS patients in real time in the future. Finally, Mayo Clinic EMR structure may be different from other institutions, thus limiting its use. The generalization of the findings is limited at this point, given that no external validation was performed. Future studies should evaluate the method in different EMR systems and in different populations.

## Conclusions

Here we reported the derivation and validation of an automated electronic search query algorithm for identifying ARDS patients according to the Berlin definition. Sensitivity and specificity approached 100% in this study and may continue to improve as processes develop around electronic notes searches, following the iterative development model previously described. The development of this type of automated search strategy is widely applicable to clinical research; it may improve the efficiency and accuracy of patient identification, thus furthering knowledge on the subjects and potentially improving outcomes. Ultimately, it may enable pragmatic research on large cohorts of patients using existing EMRs, for early and rapid identification of ARDS patients.

## Data Availability Statement

The original contributions presented in the study are included in the article/supplementary material, further inquiries can be directed to the corresponding author.

## Ethics Statement

This study was approved by the Mayo Clinic Institutional Review Board (IRB) for the use of existing medical records of patients who gave prior research authorization. Informed consent was obtained from all patients included in the study.

## Author Contributions

RK conceptualized the study. XS and TW designed the study. XS performed data analysis and drafted the manuscript. YD and KK critically revised the article for important intellectual content. All authors read and approved the final manuscript.

## Conflict of Interest

The authors declare that the research was conducted in the absence of any commercial or financial relationships that could be construed as a potential conflict of interest.
